# Towards A New Approach for the Description of Cyclo–2,4-Dihydroxybenzoate, A Substance Which Effectively Mimics Zearalenone in Imprinted Polymers Designed for Analyzing Selected Mycotoxins in Urine

**DOI:** 10.3390/ijms20071588

**Published:** 2019-03-29

**Authors:** Renata Gadzała-Kopciuch, Katarzyna Kwaśniewska, Agnieszka Ludwiczak, Piotr Skrzyniarz, Rafał Jakubowski, Wiesław Nowak, Andrzej Wojtczak, Bogusław Buszewski

**Affiliations:** 1 Department of Environmental Chemistry and Bioanalytics, Faculty of Chemistry, Nicolaus Copernicus University in Torun, Gagarina 7, Pl-87 100 Toruń, Poland; k.kwasniewska88@wp.pl (K.K.); bbusz@umk.pl (B.B.); 2 Interdisciplinary Center for Modern Technologies, Nicolaus Copernicus University in Torun, Wileńska 4, 87–100 Toruń, Poland; wiesiek@fizyka.umk.pl; 3 Department of Geobotany and Landscape Planning, Faculty of Biology and Environmental Protection, Nicolaus Copernicus University in Torun, Lwowska 1, Pl-87 100 Toruń, Poland; agnieszka.b.1987@o2.pl; 4 Institute of Physics, Faculty of Physics, Astronomy and Informatics, Nicolaus Copernicus University in Torun, Grudziądzka 5, 87–100 Toruń, Poland; piotr@skrzyniarz.com.pl (P.S.); rjakubowski@fizyka.umk.pl (R.J.); 5 Department of Crystallochemistry and Biocrystalography, Faculty of Chemistry, Nicolaus Copernicus University in Torun, Gagarina 7, Pl-87 100 Toruń, Poland; awojt@chem.uni.torun.pl

**Keywords:** zearalenone, molecularly-imprinted polymers, crystallographic analysis, molecular modeling, molecularly imprinted solid-phase extraction (MISPE)

## Abstract

A method of purifying cyclododecyl 2,4-dihydroxybenzoate as a potential replacement template molecule for preparation of molecularly-imprinted polymers for isolation of zearalenone in urine was developed. Full physicochemical characteristics of cyclododecyl 2,4-dihydroxybenzoate for the first time included crystallographic analysis and molecular modelling, which made possible the determination of the similarity between the cyclododecyl 2,4-dihydroxybenzoate and zearalenone molecules. The obtained molecularly-imprinted polymers show very high in vitro selectivity towards zearalenone due to specific interactions (e.g., hydrogen bonding, molecular recognition interaction). The achieved extraction recovery exceeds 94% at the tested concentration levels (20–500 ng·mL^−1^) with a relative standard deviation below 2%. Immunosorbents were found to have lower recoveries (below 92.5%) and RSD value between 2 and 4% for higher concentrations of the studied substance (400 ng·mL^−1^).

## 1. Introduction

Zearalenone is a mycotoxin that has many harmful effects on the health of animals and humans, in particular, hyperestrogenism [1,2]. Exposure to this xenoestrogen results in deformed genitals, disorders in the reproduction process and feminization of male animals [3,4,5]. Zearalenone is also suspected to be one of the risk factors in female genital carcinogenesis [6,7,8], and in recent years research on its carcinogenic effects on the prostate gland has also been reported [9]. The latest research suggests that zearalenone content in urine can be a potential biomarker of exposure to mycotoxin [10,11,12] present, for example, in corn products infected with *Fusarium*. However, obtaining biological material for analysis, such as plasma, bile, or tissue for research of this kind is quite difficult because sample collection is invasive. Another problem is related to the very specific conditions which must necessarily be observed during collecting, preserving, and transporting the material. Thus, measuring of zearalenone content in urine is a more practical and promising strategy [10,11,12].

The most important step in the determination of analytes in any biological matrices is the sample preparation [13,14]. The research conducted so far on isolating zearalenone in urine has focused on enzymatic hydrolysis combined with liquid-liquid extraction, miniQuEChERs, dispersive liquid–liquid microextraction (DLLME), and salting-out liquid–liquid extraction (SALLE), or with multiple dilution of samples [15,16,17,18].

There are only a few reports (Table 1) on using solid phase extraction with sorbents [19,20,21,22,23,24]. The majority of these papers use a combination of two sorbents—an initial isolation-purification with an octadecyl sorbent and a secondary purification using immunosorbents. Molecularly-imprinted polymers (MIPs), as tailor-made biomimetic materials synthesized via polymerization process in the presence of template molecules, can specifically extract target compounds from complex matrices [25,26,27,28,29,30]. Thus, there is a need to increase sample processing efficiency by combining the extraction process with a more robust purification process, which can also increase compound recovery, such as with molecular imprinted polymers (MIP). Preparation of molecularly-imprinted polymers involves the formation of a prepolymerization complex between the template molecule and a suitable functional monomer. Due to the non-covalent interactions between these compounds, a cavity for the determined analyte molecule is formed after the addition of cross-linking agents. It is desirable that an imprinted molecule should be the same as the analyzed molecule. However, in some cases a good strategy is to use a different compound as a template. This approach is beneficial when the price of the primary template is expensive, such as is the case for a zearalenone template, and high recoveries can also be obtained using a different compound as a template. The cost of ZEA is very high. Finding a good substitute would make large-scale sensitive chromatographic analysis more feasible and useful.

Urraca et al. [27] proposed three compounds that might be used for imprinting instead of ZEA: resolcinol, β-resolcylic acid and cyclododecyl 2,4-dihydroxybenzoate (CDHB). The structural fragment of ZEA that might be important for the MIP technology should include resolcylic acid with the carboxylic group. Resolcinol and β-resolcylic acid might constitute a good replacement template for MIP due to the specific interaction of the hydroxyl groups, which are necessary for the formation of a cavity accommodating the analyte molecule. It has been shown that the template based on CDHB can be efficiently used for molecularly-imprinted polymers dedicated for ZEA [28,29]. Since CDHB contains a diphenylcarboxyl moiety and the C12 aliphatic ring, its use results in the formation of cavities capable of selective binding of zearalenone. The use of these three compounds for MIP was not fully characterized by Urraca et al. [27], especially for CDHB.

The primary aim of this study was to develop purification methods after CDHB synthesis and to characterize the physico-chemical characteristics for CDHB’s suitability as a replacement molecule (template) for ZEA. To assess the structure and conformation of CDHB, radiographic structural analysis (X-ray) and molecular modeling were employed, taking into account the results obtained from standard analytical techniques, such as infrared spectroscopy (FT-IR), nuclear magnetic resonance (^1^H and ^13^C) and UV–VIS spectrophotometry. The collected results of the analysis were further verified by quantum-chemical calculations confirming that the CDHB molecule to a large extent imitates zearalenone. Another aim was to improve the purification technique for CDHB to make it more suitable as an MIP template for isolating zearalenone from lyophilized human urine.

## 2. Results and Discussion

### 2.1. Characterization of CDHB

#### 2.1.1. FT-IR Analysis

The chemical structure of the obtained CDHB was confirmed via infrared spectroscopy (FTIR) (Table 2).

#### 2.1.2. NMR

NMR ^1^H (700 MHz, CDCl_3_) δ (ppm): 1.34–1.46(m, 18H, CH_2_); 1.62–1.66 (m, 2H, CH_2_); 1.80–1.85 (m, 2H, CH_2_); 5.24-5.27 (m, 1H, CH_2_); 5.,58 (bs, 1H, OH); 6,36 (dd, *J* = 2,1, *J* = 8.4, 1H, CH_Ar_); 6.39 (d, *J* = 2,1, 1H, CH_Ar_); 7.73 (d, *J* = 8.4, 1H CH_Ar_), 11.16 (s, 1H, OH). NMR ^13^C (175 MHz, CDCl_3_) δ (ppm): 21.84 (2 CH_2_); 24.32 (2 CH_2_); 24.97 (CH_2_); 25.16 (2 CH_2_); 30.06 (2 CH_2_); 74.45 (CH); 104.04 (CH_Ar_), 107.33 (C_Ar_); 108.84 (CH_Ar_), 132.89 (CH_Ar_); 163.07 (C_Ar_), 164.04 (C_Ar_), 170.75 (COO).

#### 2.1.3. UV–VIS

λ_abs_^max^ (ε/L·mol^−1^·cm^−1^, MeOH): 226 nm (9556), 259 nm (15,443), 295 nm (6687); (ε/L·mol^−1^·cm^−1^, ACN) 220 nm (13,101), 257 nm (14,953), 295 nm (5519).

#### 2.1.4. Mass Spectrum

Mass spectrum (*m*/*z*, %): 319.1915 (100%) [M − H]^−^, 320.190 (21.1%), 321.1976 (2.91%).

### 2.2. Characterization of Polymers

The obtained polymers were subjected to spectral analysis in order to determin the structure and effectiveness of template washing. Based on the obtained solid state NMR for ^13^C, it can be concluded that the values of chemical transitions correspond to the substituents and functional groups of the functional monomer (1-ALLP; δ = 46.3; 55.2; 60.9; 117.4 i 134.2 ppm) and networking monomer (δ = 17.9; 22.9; 66.3; 125.2; 136.0; 136.0 i 167.2 ppm) used for polymerization. With respect to MIPs, we did not detect the presence of bands corresponding to chemical transitions for functional groups present in the ZEA molecule (δ = 220 i 171 ppm) or CDHB (δ od 163–166 ppm). While making an interpretation of the obtained spectra for polymers, it may be concluded that the values of chemical transitions correspond only to the monomers which they contain. These observations are confirmed by the results obtained from infrared spectroscopy (IR) (Table 2). For 1-ALLP and TRIM based ones, an intense adsorption line can be observed around 1740 cm^−1^, which represents the vibrations of carbonyl stretch groups ν (C=O). This confirms the presence of a carbonyl group in all polymers. Additionally, at the frequency of 1639 cm^−1^ there appears a line representing stretching vibrations C=C from unreacted vinyl groups; there is another at 1267 cm^−1^ (C–O irregular deformation vibrations of ester group). The results were obtained on the basis of the established low-temperature adsorption isotherms and mercury porosimetry.

### 2.3. Quantum-Chemical Calculations

**Figure 1:**
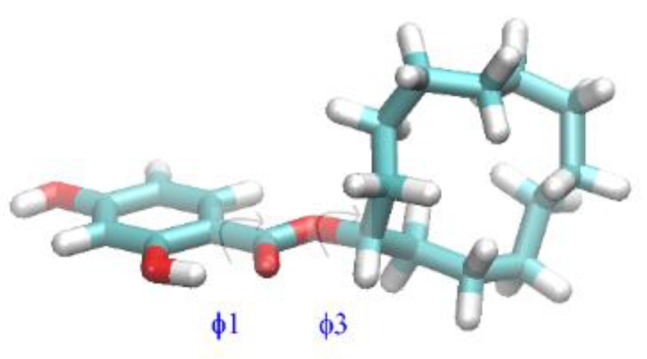
The structure of the lowest DFT energy conformer (K1) and definitions of the scanned torsional angles (φ_1_ = C2-C1-C7-O4, φ_3_ = C7-O4-C8-C9).

**Figure 2:**
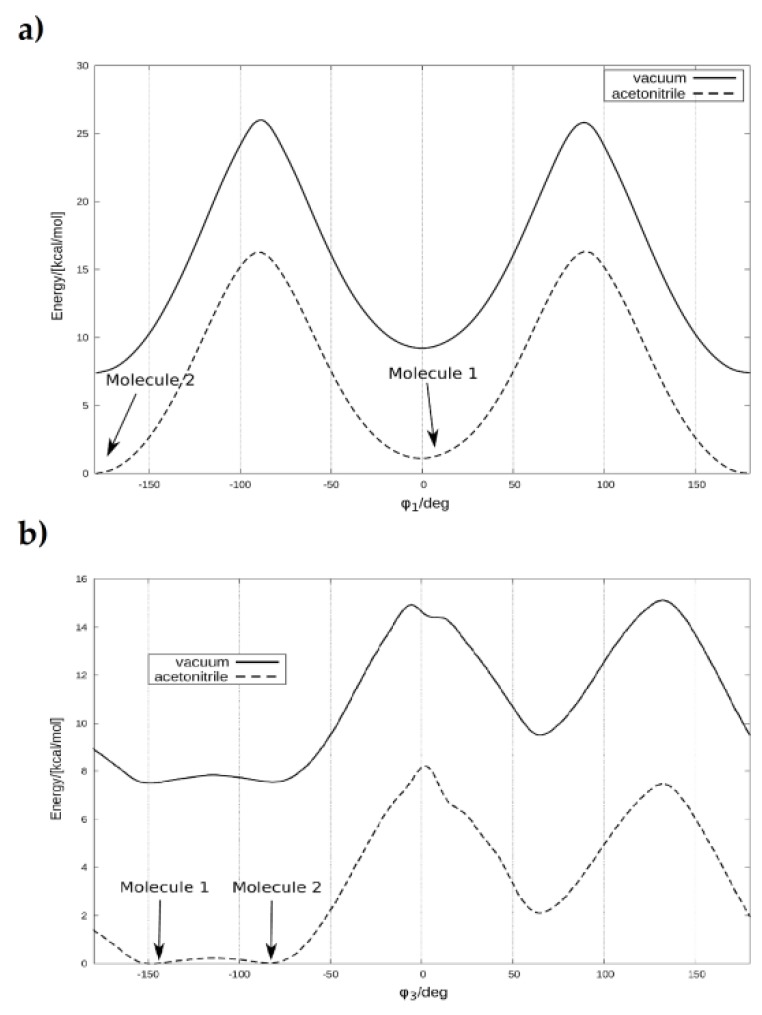
The DFT torsional potentials of CDHB for φ_1_ (**a**) and φ_3_ (**b**) coordinates. Torsional angles are defined as φ_1_ = C2-C1-C7-O4, φ_3_ = C7-O4-C8-C9, for the numbering of atoms see Table S1.

The geometry of CDHB was optimized using the DFT method with B3LYP/6-311++G** functional/basis set. The Gaussian09 code [31] was used for calculations and GaussView software for data analysis and visualization of electrostatic potential maps. Preliminary geometry optimization was performed using the PM7 method implemented in the MOPAC7 code [32]. The cyclic aliphatic ring was randomly oriented many times in order to avoid trapping in a local energy minimum. The lowest PM7 energy structures served as inputs for the full DFT geometry optimizations. Four low energy conformers have been found (K1–K4); they differ mainly by the values of φ_1_ and φ_3_ dihedral angles (for definitions see Figure 1 and Figure 2).

Table 3 shows that the DFT optimized structures are in close agreement with crystallographic data (EXP values). Selected bond angles and bond distances of conformer K1 are compared in Supplemental Table S1.

In order to establish whether conformational transitions in CDHB are possible at room temperature, DFT torsional potentials were calculated along two torsional angles φ_1_ and φ_3_ (for definitions see Figure 1 and Figure 2). In the protocol only one value of the scanned torsional angle was kept fixed while all other coordinates were fully optimized. The effects of strongly polar solvent, i.e., acetonitrile, were included using the PCM solvation method [33]. The PCM simulation of the acetonitrile environment of CDHB approximates its environment created by 1-allyl-piperazine or trimethylolpropane trimethacrylate during the MIP preparation. The results are presented in Figure 2a,b for φ_1_ and φ_3_, respectively.

Acetonitrile was selected in PCM quantum chemical DFT calculations as a generic example of a hypothetical very polar environment of the imprinted CDHB. All real polymer matrices will have non-specific interaction effects on the molecule conformations, and thus it is expected that CDHB potentials will be located between those calculated in the vacuum (no solvent) or in ACN (Figure 2). ACN lowers the energies of all structures uniformly by ca. 6 kcal/mol, as expected, and the relative energies of the conformers and the energy barriers separating them are the same as predicted in the DFT calculations in vacuum. Thus, it was inferred that conformational analysis performed for DFT data refers also to the CDHB embedded in the polymer.

**Figure 3:**
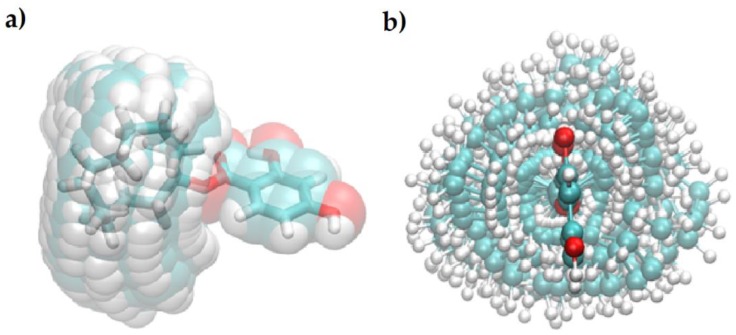
Possible shapes of cavities formed by different conformers of CDHB.

The energy barrier for the K1–K2 transition is symmetrical and for φ_1_ rotation is very low (~1 kcal/mol); thus these two conformers will be present in a solution or a crystal in equilibrium. The intramolecular H-bond to either O3 or O4 leads to such a symmetrical energy barrier for φ_1_. Those interactions were found in the crystal structure of CDHB, as described below. However, the rotation along the φ_3_ coordinate is much more restrained. The barrier between the K1 and K3 conformer of 15 kcal/mol is high (Figure 2b) and reflects the interactions between carbonyl O3 and the cyclododecyl methylene C9 or C19. The DFT calculations show the energy minima K1/K2 and K3/K4 that are consistent with the CDHB conformations of Molecule 1 and Molecule 2, respectively, as observed in the crystal structure reported here. Indeed, two such stable forms are clearly observed (Figure 2a) revealing the conformational space available for CDHB molecules and further determining the shape of the cavity formed in the MIP-CDHB (Figure 3). It should be stated that crystal packing effects may further stabilize both forms in a different orientation of the aliphatic ring, but so do the acetonitrile PCM in the calculations and the CDHB environment during the MIP preparation. It seems that such heterogeneity of CDHB is a useful feature since it provides more opportunity for binding analytes in the imprinted polymer sorbent.

A simple model of possible cavities formed by CDHB is shown in Figure 3.

**Figure 4:**
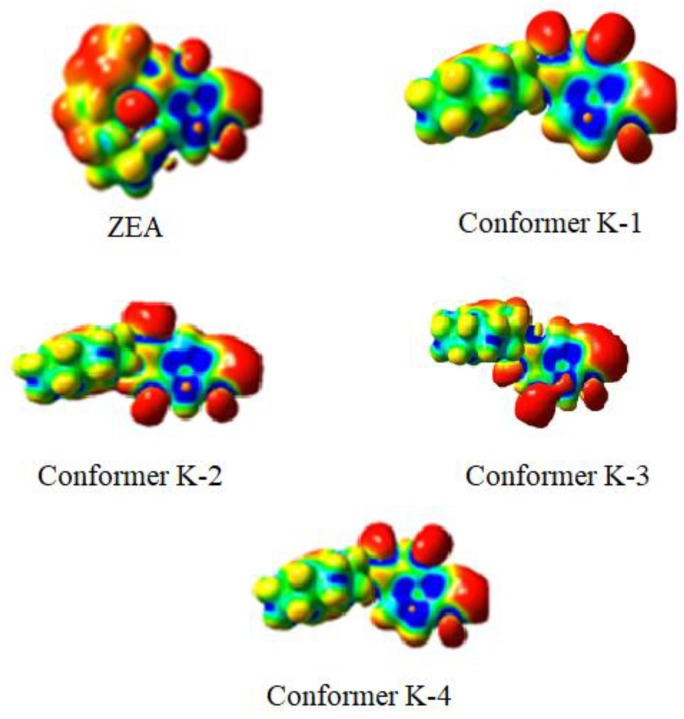
Maps of electrostatic potentials calculated using the DFT method for ZEA and CDHB (K-1 to K-4). The GaussView 4.0 code was used for visualization.

Since the plots shown in Figure 3 are based on a DFT φ_1_/φ_3_ scan of CDHB, energies of those conformers vary by at most 15 kcal/mol. At higher temperatures more and more conformers are accessible, but at 300 K a limited number of defined cavities will be formed in the polymer (Figure 4).

### 2.4. Crystal Structure of CDHB

**Figure 5:**
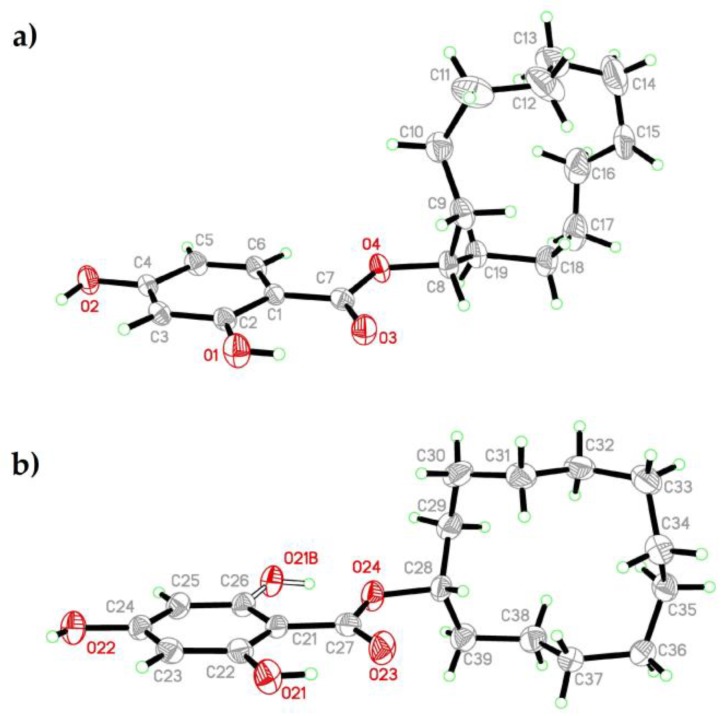
Crystal structures of CDHB: Molecule 1 (**a**), Molecule 2 (**b**).

The asymmetric part of the CDHB structure consists of two ester molecules and a single water molecule H-bonded to the esters. In Molecule 2 the aromatic ring reveals the statistical disorder with the O21 hydroxyl group occupying the alternative positions on C22 or C26, with the populations refined to approximately 73% and 27% for O21 and O21B, respectively (Figure 5b). In both orientations this hydroxyl group forms alternative H-bonds to the ester moiety O atoms. Conversely, Molecule 1 does not reveal such disorder (Figure 5a).

**Figure 6:**
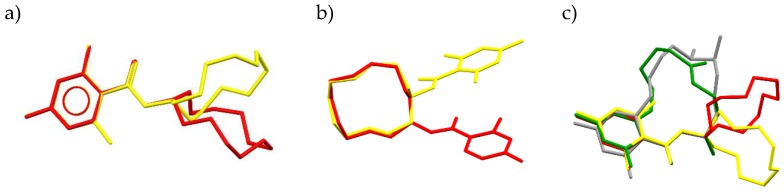
Superposition of (**a**) the aromatic ring of Molecule 1 (red) and Molecule 2 (yellow), (**b**) the cyclododecyl aliphatic ring of Molecule 1 (red) and Molecule 2 (yellow), and (**c**) the comparison of ZEA (green) and CDHB (red/yellow).

The conformation of the phenol-ester fragments in two CDHB molecules is flat (Figure 6a), with the dihedral angle between the aromatic C1--C6 ring and the best plane of the C1-C7-O3-O4 moiety or their equivalents being 8.03(3)° and 6.89(9)°, in Molecule 1 and 2, respectively. A larger difference is detected in the relative orientation of the ester moieties and the 12-membered rings, with the dihedral angles between their best planes being 89.37(5) and 80.43(6)° for Molecules 1 and 2, respectively. These differences in the relative orientation of the cyclo-dodecyl ring are further emphasized by torsion angles C7-O4-C8-C9 and C7-O4-C8-C19 and their equivalents, which are −78.63(19) and 156.41(16)°, respectively, in Molecule 1, while in Molecule 2 the corresponding values are −138.96(18) and 96.8(2)°.

In CDHB, the twist of the carboxylic moiety relative to the phenyl ring is reflected with the C2-C1-C7-O3 torsion angles being 6.9(3) and 6.0(3)° in Molecules 1 and 2, respectively. In cis-ZEA and ZEA derivative that twist is more pronounced, with the analogous angles of 17.5(6) and 16.9(5)° [33,34]. These differences are attributed to the restricted conformational flexibility of the lactone macrocycle in both ZEA structures. In both ZEA structures, the intramolecular H-bonds between the phenolic OH and carboxylate O atoms, analogous to these reported here for CDHB, also restrict the zearalenone conformation. Therefore, the resulting spatial arrangement of the molecule aromatic ring system and the polar ester moiety are similar in CDHB and ZEA molecules. There is a slight difference in the position of the carboxylic moiety relative to the aromatic ring between CDHB and both zearalenone structures.

In the CDHB structure reported here, conformation of the cyclo-dodecyl moiety seems to be different in both molecules. However, a careful analysis reveals the sequence of torsion angles identical in both molecules that can be described as p–2s-p–2s-p-5s, where p and s denote anti-periplanar and synclinal torsion angles. In contrast, a typical conformation of cyclo-dodecyl moiety as found in the CSD database (Cambridge Structural Database) [35], is described by the sequence of torsion angles p–2s-p–2s-p–2s-p–2s e.g., in [36,37]. In the reported CDHB structure, however, the superposition reveals that the same conformation of cyclo-dodecyl moiety in two molecules can be obtained with such rotation of the rings that C8 and C9 positions in Molecule 1 coincide with Molecule 2 C28 and C39, respectively. This results in the position of the ester moiety differing by one C atom of the ring (Figure 6b).

The extensive network of inter-molecular interactions is found in the CDHB structure. All donor groups, including the hydroxyl groups and a water molecule, participate in the network of the O-H…O hydrogen bonds (Table 4). In particular, the hydroxyl groups positioned ortho relative to the carboxylic moiety are involved in the intra-molecular interaction with the O atoms of this moiety. In Molecule 2 such interactions involve both disordered OH groups and are similar in their geometry. On the other hand, the additional reason for the presence of two CDHB molecules in the asymmetric unit are intermolecular H-bonds formed by the O2-H hydroxyl, which for Molecule 1 involve the O23[−x, −y+1, −z] acceptor from Molecule 2, while O22-H interacts with the water molecule. In the crystal packing of the reported structure, the C34-H34A…π interactions are found, involving the C1—C6[−x,1−y, −z] phenyl ring with the H…Cg distance to the ring center of 2.94 Å. A similar interaction C35-H35A…π involves the C1—C6[−x, −y, −z] phenyl ring and the corresponding H…Cg distance is 2.89 Å. The intra-molecular interaction C6-H6A...O4 with the C…O 2.748(2) Å is found in Molecule 1, and it is an interaction alternative to the intra-molecular H-bond involving the minor population hydroxyl in Molecule 2. Additionally, in Molecule 2 the C28-H28A...O23 interaction involving the tertiary C28 group is found, with the C…O distance of 2.748(3) Å.

Despite slightly different molecular architecture, the presence of the cyclo-dodecyl moiety in CDHB mimics, to a certain extent, the macrolide of ZEA. These similarities provide a rationale for the applicability of the CDHB template as a zearalenone model for preparing MIPs which should be efficient stationary phases for column chromatography. Comparing the molecules of zearalenone and CDHB (Figure 6c), with the latter being a proposed substitute template for ZEA, one can observe similarity in the benzene ring and only slight resemblance in the lactam ring. This may result in a lower yield as in the case of MIP-CDHB ZEA would be weaker bound to a lower degree. That might result in the lower efficiency of ZEA binding to the MIP-CDHB prepared as a less expensive alternative for MIP-ZEA. However, similarities of the intra- and inter-molecular interactions of ZEA and CDHB and the random orientation of the substitute CDHB template explains the efficiency of the CDHB-MIP system in the ZEA experiments.

### 2.5. Validation Method

The extraction and analysis parameters were optimized with a method that makes as efficient determination of analytes possible. For this aim, the efficiency of zearalenone isolation was evaluated in reference to the optimized extraction conditions, and the method for ZEA determination was validated with regard to selectivity, analytical curve, precision, accuracy, limit of detection, and limit of quantification.

#### Calibration Curves, Limits of Detection, and Quantification

Calibration curves were determined based on the analysis of standard “matrix” solutions, which were prepared on the basis of the spiked urine samples after SPE clean-up at six levels of concentration within the range 10 to 1000 ng·mL^−1^, depending on the sorbent used. For comparison purposes, calibration curves were also obtained by the analysis of sample solutions prepared in acetonitrile/water (60:40% *v*/*v*) within exactly the same concentration range as above. The collected data are presented in Table 5, which shows that the values of the coefficient of determination (*r*^2^) obtained for ZEA extracts exceed 0.99 for the majority of sorbents used. The lowest values can be observed for the linear correlation determined for ZEA extracts isolated and purified with NIP. Limits of detection and quantification were calculated based on the standard deviation of the response (δ) of the curve and the slope of the calibration curve (S) at levels approximating the LOD according to the Equations (1) and (2):

(1) $$\mathrm{LOD}\;=\;3.3\cdot \frac{\delta }{S}$$

(2) $$\mathrm{LOQ}\;=\;10\cdot \frac{\delta }{S}$$

The standard deviation of the response was determined based on the standard deviation of y-intercepts of regression lines.

### 2.6. Optimization of MISPE Parameters Influencing ZEA Adsorption

#### 2.6.1. Sample pH

**Figure 7:**
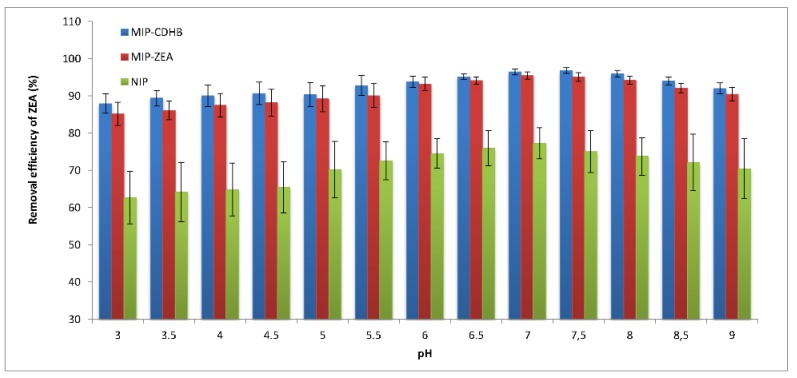
Effect of pH on the adsorption of ZEA onto MIP-CDHB, MIP-ZEA and NIP for the following adsorption conditions: mass of adsorbent = 50 mg, initial concentration = 1 mg·L^−1^, solution volume = 25 mL, T = 298 K.

The effect of the sample pH on the adsorption of ZEA on polymer sorbents: MIP-CDHB, MIP-ZEA and NIP was investigated. The sample had the initial pH = 3 subsequently incremented by 0.5 until it reached pH = 9. The results obtained are shown in Figure 7. Following the analysis of these results, it can be clearly stated that the adsorption of the isolated compound is slightly dependent on the changes in pH of the sample subjected to sorption. At low pH values (in the range of 3–5), low repeatability (high SD values) was observed in the case of ZEA sorption on both polymers with (MIP-CDHB and MIP-ZEA) and without (NIP) an imprinted molecule. For NIP, SD values were in the range from 13.8% to 15.2% while for both MIPs these values were lower (SD_MIP-CDHB_ below 6%, SD_MIP-ZEA_ 5–7.2%). In the pH range from 7.0 to 7.5, the sorption efficiency for MIP-CDHB was between 96.5 and 96.8% (SD below 2.0%), and for MIP-ZEA was from 94.8–95.5% (SD approx. 2.3%). Above pH = 8.0 there was a marginal decrease in the efficiency of ZEA removal from all polymers. However, SD values for NIP were still very high, which indicates low reproducibility of the extraction process.

#### 2.6.2. MIP Amount

**Figure 8:**
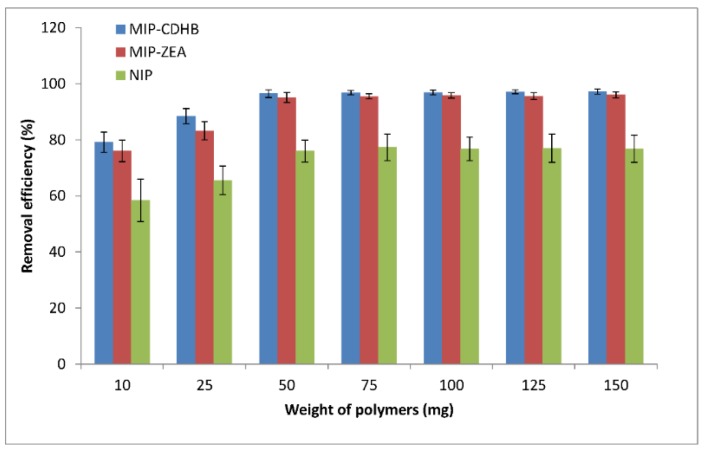
The percentage of ZEA removal at different amounts of adsorbents (MIP-CDHB, MIP-ZEA and NIP) for the following adsorption conditions: initial concentration = 1 mg·mL^−1^; solution volume = 25 mL; T = 298 K.

We investigated the influence of the amount of the sorbent on ZEA sorption by gradually increasing the sorbent amount from 10 to 150 mg at constant conditions of pH, concentration, and contact time. Figure 8 shows this relationship. It can be seen that removal of ZEA from MIP increases with increasing amounts of adsorbent. For sorbent amounts ranging from 50 to 150 mg, similar removal values were obtained (RE_MIP-CDHB_ approx. 97%; RE_MIP-ZEA_ ca. 95%). This increase is related to the progressively larger surface available for ZEA sorption due to the growing number of selective sites in MIP. At 10 and 25 mg of adsorbent we observed low percentage values of the removal; for MIP-CDHB they were respectively 79.1% and 88.4%, and for MIP-ZEA 76.0% and 83.2%. Similarly, in the case of NIP, low values of 58.4% and 65.5% were obtained. Sorption reproducibility for these two amounts of adsorbent was also low (high SD values).

#### 2.6.3. Desorption

**Figure 9:**
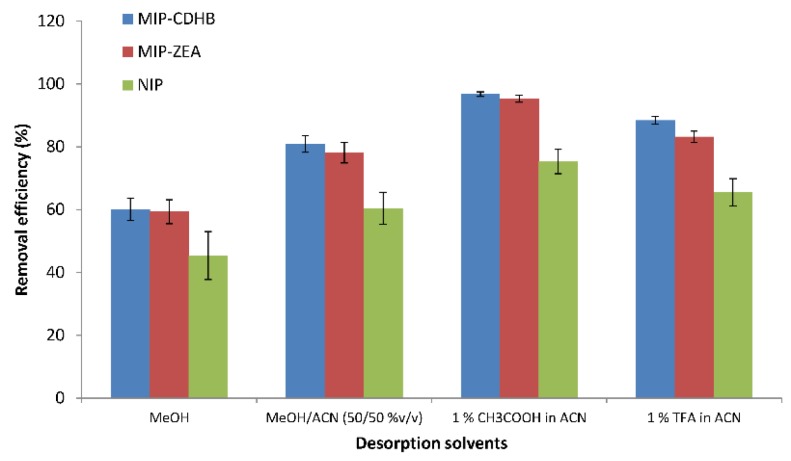
Effect of pH on the adsorption of ZEA onto MIP-CDHB, MIP-ZEA and NIP for the following adsorption conditions: adsorbent weight = 50 mg; initial concentration = 1 mg·L^−1^; solution volume = 25 mL; T = 298 K, time = 60 min.

Liquid desorption was employed in this study to desorb ZEA from the MIP. Four solvents (acetonitrile (ACN), acetonitrile and methanol (50/50% *v*/*v*), 1% acetic acid, or 1% trifluoroacetic acid in acetonitrile) were investigated as desorption solvents to break the hydrogen bonds between ZEA and 1-ALPP. The results are shown in Figure 9. As can be seen, a 1% solution of acetic acid in acetonitrile provided the best desorption of ZEA from MIPs (RE_MIP-CDHB_ = 96.8%, RE_MIP-ZEA_ = 95.5%). Selective extraction is based on the interaction of the amino groups contained in 1-ALPP and a hydroxyl group in ZEA; therefore breaking these bonds with the use of solvents such as acetonitrile or a mixture of acetonitrile-methanol (50/50% *v*/*v*) proved to be hardly effective (removal efficiency from about 60–80% for MIPs, for NIP from 45–60%). In cases where acetic or trifluoroacetic acid was added to acetonitrile, higher percentage of ZEA removal was observed for MIPs, with high process repeatability (SD below 2%).

#### 2.6.4. Kinetic Study

**Figure 10:**
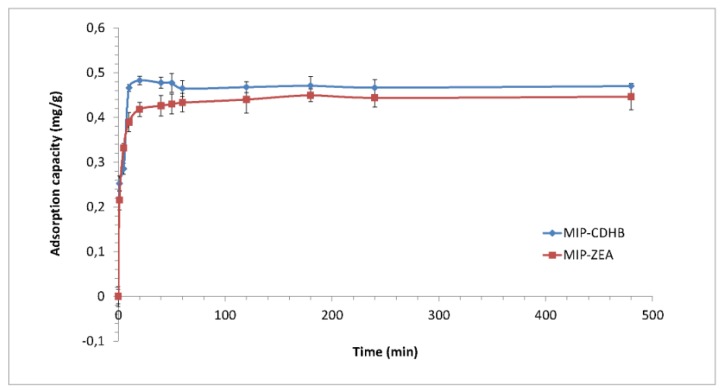
Zearalenone adsorption capacity of MIP-CDHB and MIP-ZEA.

The ZEA adsorption rate by MIP was measured as a function of time. The result of this kinetic study is shown in Figure 10. The process of ZEA sorption was fast during the initial stage and slow at the approach to equilibrium. The maximum adsorption occurred after 20 min at the capacity approx. 0.48 mg/g for MIP-CDHB, and after one hour for MIP-ZEA, where the adsorption capacity was about 0.44 mg/g.

The Lagergren equation is a commonly used rate equation in liquid phase sorption [38]. The linearized form of the pseudo-first and pseudo-second order equations was used to describe the sorption rate of zearalenone from aqueous solution on MIPs. In order to determine this relationship, the equations included in the literature [38] were used, and the results—both experimental and theoretically calculated—for the adsorption capacity at equilibrium (*q*_e_) values and coefficients related to kinetic plots are listed in Table 6. The obtained values *q*_e_ for MIPs are close to each other and different from the experimental values *q*_e_ kinetic models for the second and first row. Therefore, the second-order kinetics best describes the data better than the first-order pseudo-model.

#### 2.6.5. Adsorption Isotherm

**Figure 11:**
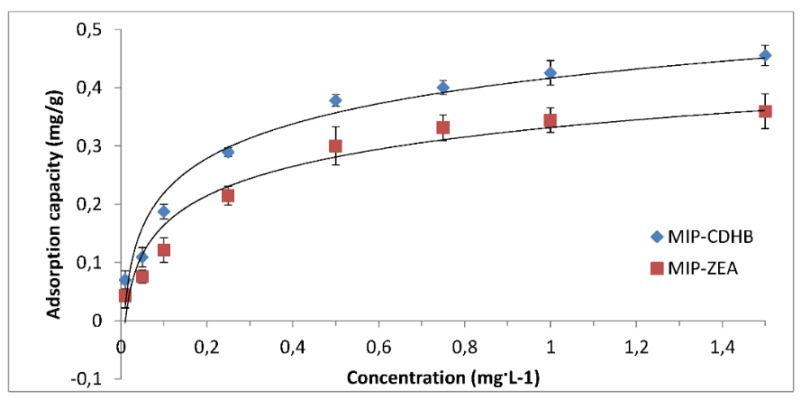
Adsorption capacity of various concentrations of zearalenone by MIP-CDHB and MIP-ZEA.

The amount of adsorbed material is determined depending on the concentration at a constant temperature, which can be represented as adsorption isotherms. Adsorption isotherm was determined for zearalenone by using MIPs under optimum conditions, and the results of these measurements are summarized in Figure 11. The maximum adsorption capacity was 0.46 mg/g for MIP-CDHB and 0.35 mg/g for MIP-ZEA. The equations used to describe the experimental data on isotherms were those developed by Langmuir and Freundlich [38], commonly used for this purpose.

#### 2.6.6. Selectivity 

The selectivity of the developed method was determined based on the results of the chromatographic analysis with fluorimetric detection dedicated to determine zearalenone in the presence of other sample components. The data presented in Table 5 were also used to calculate the matrix effect (ME) from the relation described by Equation (3) [39,40]:

(3) $$\%\;\mathrm{ME}=\;\frac{m_{std}-m_{ur}}{m_{std}}\cdot 100\%$$

where: m_std_ is the slope obtained by dosing zearalenone solutions prepared in mobile phase, and m_ur_ is the slope obtained from the calibration curve for ZEA extracts prepared in the spiked urine samples.

The calculated matrix effect for ZEA extracts isolated with MIP-ZEA equals 19.7%; for MIP-CDHB – %ME = 15.1%; for NIP – %ME= 38.1%; for ImmunoClean C ZON %ME = 24.4%, and for ImmunoClean C+ ZON – %ME = 19.4%. It is assumed that if the %ME is lower than 20% or greater than −20%, it can be said that matrix effects do not influence the analysis by the amount of the tested substance. It can be noted that only if extraction has been carried out with NIP, %ME clearly exceeds 20%, what suggests that the matrix can significantly impede the determination of ZEA. Slightly larger %ME can be observed for ImmunoClean C ZON. This can result in exceeding the limit of the sample volume put on the sorbent, which was suggested by the manufacturer as lower than 10 mL.

### 2.7. Effectiveness of Isolating ZEA from Urine

**Figure 12:**
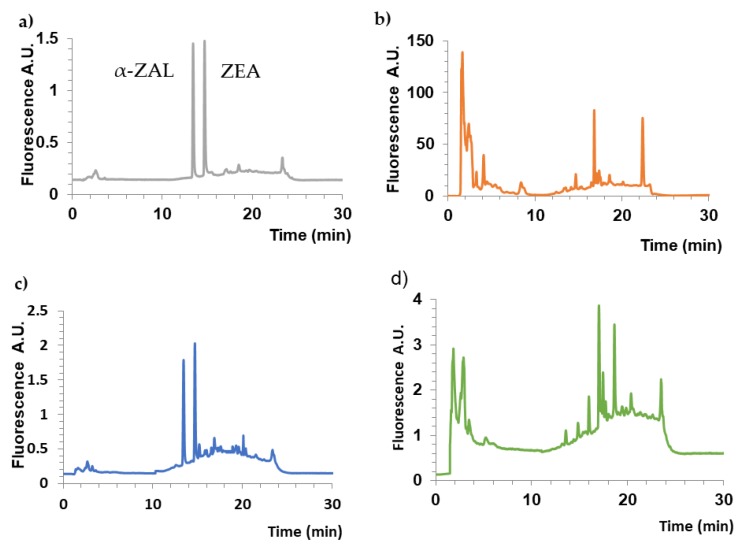
Chromatograms obtained for the standard solutions of zearalenone (ZEA) and its metabolites (α-zearalenol, α-ZAL) (**a**), untreated urine samples (**b**), MIP-CDHB (**c**), and NIP (**d**) extraction of urine spiked of 100 ng·mL^−1^ of each analyte.

Solid phase extraction using molecularly-imprinted polymers (MISPE) and immunosorbents (ImmunoClean C+ ZON, ImmunoCleanC ZON) was used as a method of isolating and enriching zearalenone as well as purifying the matrix from contaminants. In order to compare the extraction process and determine its efficiency for polymers with imprinted molecules of CDHB (MIP-CDHB) and ZEA (MIP-ZEA), the studied compound was also isolated on non-imprinted polymers (NIP) (Figure 12).

The extraction process was carried out following the procedure described above. This method ensures that ZEA is isolated from urine samples with the yield between 94–98%, with standard deviation not exceeding 2% for MIP-CDHB and MIP-ZEA polymers (Table 7). It has to be emphasized that the efficiency of MIP-CDHB used for the solid phase extraction of ZEA is identical with that of specific MIP-ZEA (within 1σ). For both MIPs, the extraction efficiency is high and the extraction repeatability is very high, which can be proven by low RSD values. As the recovery values are high, the CDHB molecule can be successfully used as an alternative template for ZEA. Considering the high price of ZEA, using a cheaper solution such as CDHB leads to lowering of the unit cost of the extraction process, even in comparison to expensive antibodies used only once.

For non-imprinted polymers, the yield values were lower than those presented above (R between 72–80%) and the repeatability is burdened with high uncertainty (RSD ca. 10%). Such high RSD values indicate lack of process repeatability, which determines the use of this sorbent as suitable for ZEA isolation. For ImmunoClean C+ ZON and ImmunoClean C ZON the yield values were between 86–92%, while RSD did not exceed 5.2%. An analogy can be noticed for all extractions: for lower concentrations, higher yield values were obtained with greater repeatability. An increase in analyte concentration in the sample resulted in lower yield values and much lower repeatability. The highest degree of ZEA purification from matrix was achieved for polymers molecularly imprinted with ZEA and CDHB particles, at the level comparable to ImmunoClean C+ZON antibodies. It should be considered that antibodies are single-use sorbents, while it was confirmed, that MIPs can be used several times.

## 3. Materials and Methods

### 3.1. Reagents and Materials

Zearalenone (ZEA), 2,2′-azobisisobutyronitrile, 1-allyl-piperazine (1-ALPP) (96%), trimethylolpropane trimethacrylate (TRIM; technical grade), cyclododecanol, 1,1′-carbonyldiimidazole (≥90%), dimethyl fumarate, 2,4-dihydroxybenzoate acid (97%), and 1,8 diazabicyclo[5.4.0]undec-7-ene were purchased from Fluka (Sigma-Aldrich Chemie, Steinheim, Germany). For MISPE and chromatographic analyses, organic solvents of high performance liquid chromatography (HPLC) grade were purchased from J.T. Baker (Groß-Gerau, Germany); organic solvents used for purification of CDHB and polymers—methanol, n-hexane, petroleum ether, and ethyl acetate—came from POCh, (Gliwice, Poland); deionized water was obtained at our laboratory with the Milli-Q system (Millipore, El Paso, TX, USA). Aluminum oxide (150 mesh), silica gel (230–400 mesh) and HPTLC-Alufolien Kieselgel 60F254 for CDHB purification were purchased from Merck (Darmstadt, Germany). Anhydrous sodium sulfate (POCh, Gliwice, Poland) was the drying agent.

Solid-phase extraction was performed using a 12-port vacuum manifold supplied by Mallinckrodt Baker (Deventer, The Netherlands). Molecularly imprinted solid-phase extraction (MISPE) columns were prepared using solid-phase extraction (SPE) glass columns (volume of 1 mL) equipped with porous polytetrafluoroethylene (PTFE) disks (Mallinckrodt Baker, Phillipsburg, NJ, USA) at the top and at the bottom of the polymer bed (50 mg).

To compare sorption effectiveness, immunoactive columns were used: ImmunoClean C+ ZON and ImmunoClean C ZON with a concentration of antibody 1 mg/mL (Aokin AG, Berlin, Germany). The study of ZEA sorption effectiveness was performed with the use of lyophilized freeze dried powdered human urine (Urinorm and Uripath, Delfzijl, the Nederlands).

### 3.2. Chromatographic Conditions

Zearalenone was determined with liquid chromatography (Model 1100, Agilent Technologies, Germany) with a fluorometric detector (Model 1260, Agilent Technologies, Germany). ZEA was chromatographed using a XBridge C18 column (150 × 4.6 mm; 3.5 μm) and a gradient separation using acetonitrile (ACN)/water in gradient elution: 0–5 min 50% ACN; 20 min 100% ACN; 30 min 100% ACN; 35 min 50% ACN. The flow rate was 0.5 mL·min^−1^. The column temperature was 25 °C. The identification was carried out at λ_Ex_ = 270 nm and λ_Em_ = 452 nm.

### 3.3. Synthesis

The synthesis of CDHB was performed following the procedure of Uracca et al. [27]. A mixture of 1,1′-carbonylodiimidazole (10 mmol) and 2,4-dihydroxybenzoic acid (10 mmol) was dissolved in 20 mL of anhydrous DMF and stirred for two hours at 40 °C. Then cyclododecanol (12 mmol) and DBU (12 mmol) were added and the mixture was stirred for 42 h. After that time 20 mL CH_2_Cl_2_ and 20 mL H_2_O were added. The organic layer was washed twice with 30 mL of 20% HCl and 30 mL of saturated NaCl solution.

The organic layer was dried with anhydrous Na_2_SO_4_. Next, the crude product was purified with silica gel and the eluent composed of petroleum ether:ethyl acetate 6:1 (*v*/*v*) was used. The fractions then were collected in glass tubes and analyzed by thin layer chromatography using a mixture of n-hexane:ethyl acetate 1:1 (*v*/*v*). The fractions containing pure CDHB were evaporated and allowed to dry in a vacuum oven at the temperature of 60 °C for 24 h. The white solid was recrystallized from acetonitrile-water mixture to obtain colourless crystals.

### 3.4. Polymer Synthesis: MIP and NIP

**Figure 13:**
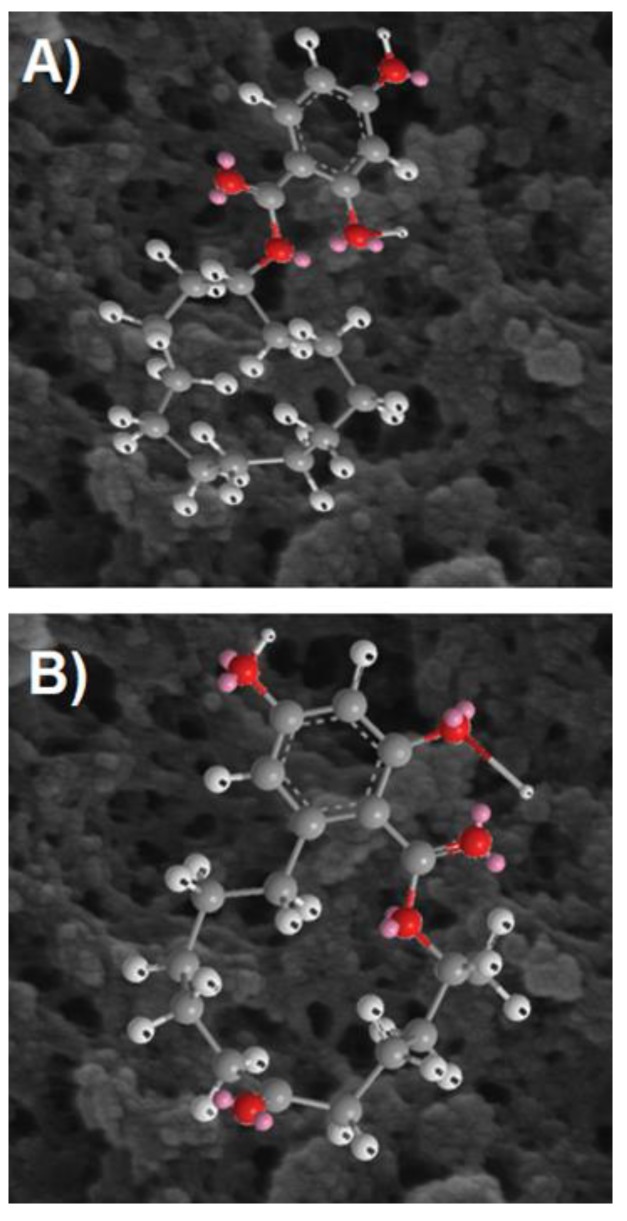
CDHB (**a**) structural similarity to ZEA (**b**) in the molecularly imprinted polymer on the background of images (×100,000) obtained by scanning electron microscopy (SEM).

Molecularly-imprinted polymers were obtained following the previously established and described procedure [27,29], using as monomers 1-allyl-piperazine (1-ALPP), trimethylolpropane trimethacrylate (TRIM) and CDHB or zearalenone (MIP-ZEA) (Figure 13). Non-imprinted polymer (NIP) was synthesized similarly to the MIP, but without the template molecule. Before packing the sorbents into columns, the remaining monomers and template were removed from the polymers through Soxhlet extraction (24 h at 20 cycles/h) with a mixture of methanol/acetic acid (96/4% *v*/*v*). The polymers were dried in vacuum for 24 h at 45 °C and subsequently crushed and sifted into fractions (30–60 μm). The dried polymers (50 mg), suspended in 5 mL of methanol, were transferred into 1 mL SPE glass cartridges (J.T. Baker Chemical Co., Phillipsburg, NJ, USA) capped with two polytetrafluoroethylene (PTFE) frits at each end.

### 3.5. Characterization of CDHB crystals

The purified crystalline product was characterized using an FT-IR Spectrum 2000 in the frequency range 4000–400 cm^−1^. NMR analysis was performed using a Bruker Avance III 700 MHz machine. The UV–VIS spectrum (HELIOS alpha, UNICAM, Labsoft, Warsaw, Poland) was collected between 190–600 nm. Compound purity was assessed using a high-resolution tandem spectrometer Q-TOF/MS (Agilent G6540B, Agilent Technologies, Santa Clara, USA). X-ray diffraction data for CDHB monocrystals were collected using an Oxford Sapphire CCD diffractometer using MoKα radiation λ = 0.71073 Å, at 293(2) K, by ω–2θ method. Based on the systematic absences, the space group was determined as monoclinic P2_1_/n. The structure was solved by direct methods and refined with the full-matrix least-squares method on F^2^ with the use of SHELX2014 [41] program package. The analytical absorption correction was applied (Table 8). Positions of hydrogen atoms were found from the electron density maps, and hydrogen atoms were constrained in the refinement with the appropriate riding model as implemented in SHELX. The X-ray experimental data and structure refinement for the reported structure are summarized in Table 8. The structural data have been deposited with The Cambridge Crystallographic Data Centre, the deposition number CCDC XYZ. The data can be obtained free of charge from The Cambridge Crystallographic Data Centre via www.ccdc.cam.ac.uk/structures.

### 3.6. Characterization of Polymers

#### 3.6.1. Physicochemical Characterization of Polymers

In order to confirm the created structure of the obtained polymers, various physicochemical techniques were applied, including mercury intrusion porosimetry (ASAP 2012, Micromeritics Instrument Corp., Norcross, USA), infrared spectroscopy (Perkin-Elmer 1800 (Norwalk, CT, USA), nuclear magnetic resonance in the solid state (Bruker 300 MSL, Rheinstetten, Germany), and scanning electron microscopy (SEM) (Figure 13) (Leo 1430 VP, Electronenmikroskopie, GmbH, Oberkochen, Germany). The development of these techniques increases chances for precise determination of the surface structure of the obtained polymers. Low-temperature adsorption-desorption of nitrogen was used to establish isotherms of Brunauer-Emmett-Teller (BET) adsorption, based on which the specific surface area (m^2^·g^−1^) of the obtained sorbents was determined. Based on the Barrett-Joyner-Halenda (BJH) method, the pore volume (mL·g^−1^) and pore diameter were calculated (Table 9). Nitrogen adsorption is used mainly for the analysis of micro- and mesopores, due to the size of nitrogen molecules. Comparing the imprinted and the non-imprinted polymer, it can be concluded that MIPs have a higher specific surface area, pore volume and pore diameter than NIPs. These results directly indicated a significant influence of the template on the polymer structure, where the presence of imprinted cavities increased the surface area and provided a higher number of selective binding sites [38,42].

#### 3.6.2. Adsorption Studies

The experiments investigated the adsorption of ZEA from aqueous solutions, which included determining the effect of such factors as MIP amount, pH, adsorption isotherms, kinetics and selectivity of the obtained MIP. The final concentration of ZEA (C_e_) was determined using HPLC-FLD analysis (on chromatographic conditions, see Section 3.2).

The preliminary studies were aimed at the selection of a suitable solvent for the sorption of ZEA and contact time required to reach equilibrium. 50 mg of MIP was added to a solution of ZEA with initial concentrations (1 mg·L^−1^) pH neutral and shaken at 23 °C.

For the pH study, 50 mg of MIP was shaken for 30 min in 1 mg·L^−1^ ZEA (25 mL). The pH (pH range 3–9) was adjusted with hydrochloric acid or sodium hydroxide.

Initial studies were also carried out to determine the best selective solvent for ZEA desorption and the contact time required to achieve equilibrium. Fifty milligrams of MIP was added to the solution of an initial concentration of ZEA (1 mg·L^−1^) at natural pH and shaken at 23 °C.

The adsorption kinetics was studied by stirring MIP (50 mg) in 1 mg·L^−1^ ZEA (25 mL) for different amounts of time (5, 15, 25, 45 min and 1, 2, 3 h). The adsorption isotherms were studied by stirring MIP (50 mg) in ZEA solution (25 mL) at different concentrations: 10, 50, 100, 250, 500, 750, 1000 and 1500 ng·mL^−1^. The concentration of ZEA in the aqueous solutions remaining after sorption was analyzed by HPLC-FLD. The ZEA uptake at equilibrium defined as the adsorption capacity (*q*) and the percentage of removal efficiency (*RE*) were calculated by using Equations (4) and (5) respectively:

(4) $$q=\frac{\left(C_{0}-C_{e}\right)\cdot V}{m}$$

(5) $$\mathrm{RE}=\;\frac{\left(C_{0}-C_{e}\right)}{C_{0}}\cdot 100\%\;$$

where *q* (mg/g) is the amount of total adsorption of ZEA, *C*_0_ and *C*_e_ are initial and equilibrium concentration of ZEA in solution (mg·L^−1^), *V* (L) is the volume of the solution and *m* (g) is the weight of MIP.

#### 3.6.3. Binding Analysis of Molecularly-Imprinted Polymers

For research of the ZEA sorption on the obtained polymers, a model solution of lyophilized urine with addition of acetate buffer (pH = 4.8) and PBS (pH = 7.4) (in proportions in accordance with the description in Section 2.7) which was contaminated with standard solution of zearalenone so that the final concentration was 100 ng in 1 mL of this solution. Twenty milligrams of the sorbent were weighted and placed into 2 mL Eppendorf tubes and 1 mL of model urine solution was added. The tubes were then shaken (150 rpm) in a heated bath (room temperature) for three hours and next centrifuged for five minutes (10,000 rpm). The concentration of ZEA was determined based on the measurements using HPLC-FLD. The amount of ZEA bound to the polymer was calculated based on its concentration in MIP samples with NIP samples, where Q_MIP_ and Q_NIP_ are the amounts of bound ZEA on the polymer with imprinted template and without an imprinted template, respectively. The ligand binding efficiency (α) was calculated as a percentage proportion of the compounds bound by MIP to the percentage of the analytes bound by NIP. The results (Table 10) confirm that MIP have a higher zearalenone adsorption ability compared to ZEA adsorption on NIP. The imprinting sites formed in MIP have an ability to distinguish target molecules by their size, shape and distribution of functional groups [43,44]. However, NIP adsorbs only ZEA on the surface due to the lack of imprinting sites in the polymer network.

### 3.7. Urine Sample Preparation

Lyophilized urine was dissolved in 5 mL of double distilled water and 10 mL of acetate buffer with pH = 4.8 was added to the solution. The samples were mixed on a Vortex shaker (1 min). Samples of four levels of ZEA concentration (20, 100, 400, and 500 ng·mL^−1^) were simultaneously prepared and incubated in a bath (37 °C, 18 h) with added β-glucoronidase/sulfactase (100 μL). The samples were centrifuged for 5 min (1000 rpm; 123× *g*). Twenty milliliters of PBS (pH = 7.4) was added to the clear solution, and the mixture was transferred quantitatively to the extraction columns. Polymer sorbents had been conditioned with 3 mL of methanol, 3 mL of double distilled water, and 3 mL of PBS (pH = 7.4), while the immunoaffinity sorbents had no previous preparation. The liquid flow rate through the sorbent was 1 mL·min^−1^. The columns were washed with 5 mL of PBS solution with 10% methanol. The elution was carried out twice with 1 mL of 100% methanol for the ImmunoClean C ZON and ImmunoClean C+ ZON columns; for polymers (MIP-CDHB, MIP-ZEA and NIP) 1% acetic acid in acetonitrile (two 500 μL portions) was used. The extract was evaporated at 40 °C in a stream of inert gas (nitrogen) and the dry matter was dissolved in 500 μL of acetonitrile/water (60:40% *v*/*v*) used in the liquid chromatography. The samples then were subjected to the HPLC analysis.

### 3.8. Validation Procedure

To ensure the quality of the obtained results, validation of the developed analytical methodology was planned and carried out, based on reference guide documents published internationally [45,46,47] and accessible literature [39,40]. It was shown that the method can be validated for the planned purpose by evaluating the following parameters: calibration curves, limits of detection (LOD), limits of quantification (LOQ), selectivity, precision, and matrix effects.

## 4. Conclusions

The proposed method of CDHB purification turned out to be highly efficient in comparison to those previously used and described in the literature. The developed method of CDHB purification results in a higher purity of the substance and, thus, makes it possible to obtain MIPs of much higher selectivity than the immunosorbents. The results of this study allow us to conclude unequivocally that the CDHB molecule can be an efficient replacement molecule for zearalenone in the synthesis of molecularly-imprinted polymers. The full characteristics of this compound explained the similarities between CDHB and ZEA, which constitute the molecular basis for the application of CDHB as a substitute template for ZEA Results of the sorption process study confirmed this concept: the effectiveness of MIP-CDHB was the same as that of MIP-ZEA. The obtained molecularly-imprinted polymers (MIP-CDHB and MIP-ZEA) had much greater affinity for the isolated molecule, with high repeatability of the process. Therefore, MIPs can be successfully used as alternative sorbents in solid phase extraction, since in contrast to immunosorbents they are much less expensive materials, with preserved high repeatability. Moreover, the developed procedure shows high effectiveness in purifying ZEA from interfering substances present in urine—low values of % matrix effect.

## Figures and Tables

**Table 1:** The effectiveness of ZEA sorption from urine by various adsorbents.

Adsorbent	Recovery (SD) (%)	Methods	LOD/LOQ	Concentration Range	Subjects	Ref.
ELISA	not specified	LC-MS/MS	^1)^0.02/0.007 mg·kg^−1^	0.3–100 ng·mL^−1^	Pigs	[11]
RP C18 Phenomenex	96.6 (3.8)	LC-MS/MS	0.1/0.5 pg	0.5–100 pg	Cow, pigs	[19]
ISOLUTE^®^ C18/immunoaffinity column Easi-Extract^®^ Zearalenone	108.2 (7.5)	HPLC–APCI–MS	0.1/0.5 μg·L^−1^	0.5–100 μg·L^−1^	Horse	[20]
^2)^LLE/BakerBond C18 and NH_2_	94.3–114.0 (9–19.8)	LC-MS/MS	Detection limit: 0.11 μg·L^−1^	0.18–5 μg·L^−1^	Animal	[21]
SAX SPE cartridge	not specified	LC-MS/MS	LOD: 61 pg·mL^−1^ α-ZEL; 117 pg·mL^−1^ β-ZEL14GlcA	-	Human	[22]
Supelco Titan C18	not specified	UHPLC-MS/MS	LOD: 0.31 μg·L^–1^ ZEA; 0.11 μg·L^−1^ α-ZEL	-	Pregnant women	[23]
C18	94.3	LC-ESI/MS/MS	0.02/0.05 ng·ml^−1^	1.81 pg·mg^−1^ Creatinine	Mares	[24]
MIP-CDHB	95.2–98.2	HPLC-FLD	^3)^LOQ: 5.4 ng·mL^−1^	10–1000 ng·mL^−1^	Lyophilized human urine	[this work]
	(1.5–2.0)					
MIP-ZEA	94.1–97.1		6.3 ng·mL^−1^			
	(1.6–1.9) ^4)^					
NIP	72.1–80.0		36.9 ng·mL^−1^	50–1000 ng·mL^−1^		
	(8.2–11.2) ^4)^					
ImmunoClean C ZON	86.5–92.5		10.8 ng·mL^−1^	15–500 ng·mL^−1^		
	(2.3–5.2) ^4)^					
ImmunoClean C+ ZON	88.2–92.1		8.3 ng·mL^−1^			
	(1.9–3.8) ^4)^					

^1)^ LOD and LOQ in relation to the diet and not to the urine; ^2)^ LLE—liquid-liquid extraction; ^3)^ the parameter determined for ZEA isolated from the matrix; ^4)^ the relative standard deviation.

**Table 2:** The IR regions of the spectrum.

Wavenumber (cm^−1^)	Frequency Assignment
3402	O-H stretching, and possibly intra molecular hydrogen bonded –OH groups
3200	O-H stretching, and possibly intra molecular hydrogen bonded –OH groups
2953	C-H stretching
1686	C=O stretching
1469	C-C stretching in aromatic ring
1255	C-O stretching
850	C-H out of plane

**Table 3:** Calculated values of energy, electric dipole moments and torsion angles for particular conformers. Reference total energy of K1 contains ZPE correction (+0.4 au).

Conformer	Energy (kcal/mol, [au])	Dipole Moment (Debye)	φ_1_ (°)	EXP φ_1_ (°)	φ_3_ (°)	EXP φ_3_ (°)
K1	0.00 [−1041.672265]	2.00	179.97	−172.1	−149.53	−138.96
K2	3.28	1.10	0.18	6.03	−149.44	−139.96
K3	4.98	4.05	179.92	−172.1	−83.79	−78.63
K4	7.85	1.50	0.56	6.03	−83.79	−78.63

**Table 4:** Hydrogen bonds in CDHB molecules (given in Å and °).

	D-H	A	d(D-H)	d(H...A)	d(D...A)	<(DHA)
Mol 1	O1-H101	O3	0.82	1.9	2.6218(17)	145.3
	O1W-H1W1	O3[x−1/2, −y+3/2, z−1/2]	0.95	1.90	2.8449(18)	174.2
	O2-H1O2	O23[−x, −y+1, −z]	0.82	1.96	2.7820(18)	175.5
Mol 2	O21-H21A_a	O23	0.82	1.93	2.635(2)	143.4
	O21B-H21B_b	O24	0.82	1.83	2.528(5)	141.9
	O22-H22O	O1W	0.82	1.84	2.6596(19)	174.8
	O1W-H2W1	O22[−x−3/2, y+1/2, −z−1/2]	0.94	1.91	2.8393(17)	168.7

**Table 5:** Analytical curves (HPLC-FLD), limits of detection and quantification for ZEA in the matrix (urine).

Type of SPE Sorbent	Range of Concentration (ng mL^−1^)	Calibration Equation y = mx ± b	*r* ^2^	LOD (ng mL^−1^)	LOQ (ng mL^−1^)	% ME
MIP-CDHB	10–1000	y = 0.0254x + 0.6721	0.9990	1.8	5.4	15.1%
MIP-ZEA		y = 0.0240x + 0.4383	0.9985	2.1	6.3	19.7%
NIP	50–1000	y = 0.0185x + 1.7157	0.9815	11.2	36.9	38.1%
ImmunoClean C ZON	15–500 ^*)^	y = 0.0226x – 0.0549	0.9918	3.2	10.8	24.4%
ImmunoClean C+ ZON		y = 0.0241x + 0.8189	0.9990	2.5	8.3	19.4%

^*)^ If immunosorbents are used, the recommended amount is below 500 ng ZEA as it was observed for larger values that the recovery was reduced and the matrix effect increased.

**Table 6:** Second order kinetic results for adsorption of ZEA by MIPs.

Type of SPE Sorbent	t/q_e_ = const∙t	*r* ^2^	*q*_e_
MIP-CDHB	t/q_e_ = 2.2454·t	0.999	1.8
MIP-ZEA	t/q_e_ = 2.1311·t	0.999	2.1

**Table 7:** Effectiveness of zearalenone extraction from contaminated urine at four concentration levels when MISPE and immunoassay columns are used. Recoveries (R) and relative standard deviation (RSD) calculated from six replicates.

Sorbent	ZEA Concentration							
	20 ng·mL^−1^		100 ng·mL^−1^		400 ng·mL^−1^		500 ng·mL^−1^	
	R (%)	RSD (%)	R (%)	RSD (%)	R (%)	RSD (%)	R (%)	RSD (%)
MIP-CDHB	98.2	1.5	97.3	1.9	95.2	2.0	96.5	1,8
MIP-ZEA	97.1	1.6	96.5	1.7	94.1	1.9	95.1	1.9
NIP	72.1	9.6	80.0	8.2	78.1	10.3	75.2	11.2
ImmunoClean C ZON	90.0	4.5	92.5	2.3	90.1	4.3	86.5	5.2
ImmunoClean C+ ZON	91.8	3.2	92.1	1.9	91.4	2.2	88.2	3.8

**Table 8:** Crystal data and structure refinement for CDHB.

Empirical Formula	C_38_H_56_O_9_
Formula weight	656.83
Temperature; K	293(2)
Wavelength; Å	0.71073
Crystal system, space group	Monoclinic, P2_1_/n
Unit cell dimensions; Å and °	a = 17.1943(7)
	b = 8.1870(4)
	c = 25.8661(11)
	beta = 92.282(4)
Volume; Å^3^	3638.3(3)
Z, Calculated density; Mg/m^3^	4, 1.199
Absorption coefficient; mm^−1^	0.084
F(000)	1424
Crystal size; mm	0.45 × 0.26 × 0.13
Theta range for data collection	2.37 to 28.15º
Limiting indices	−22 ≤ h ≤ 22, −9 ≤ k ≤ 10, −34 ≤ l ≤ 31
Reflections collected/unique	23663/7895 [R(int) = 0.0524]
Completeness to theta	26.00 99.9%
Max. and min. transmission	0.9902 and 0.9667
Refinement method	Full-matrix least-squares on F^2^
Data/restraints/parameters	7895/0/434
Goodness-of-fit on F^2^	0.779
Final R indices [I > 2sigma(I)]	R1 = 0.0420, wR2 = 0.0805
R indices (all data)	R1 = 0.1415, wR2 = 0.0978
Largest diff. peak and hole; e.A^−3^	0.214 and −0.147

**Table 9:** Specific surface areas (*S_BET_*), total pore volumes (*V*_p_), and mean pore diameters (*d*_p_) for synthesized NIP and MIPs.

Polymer Code	*S*_BET_ (m^2^·g^−1^)	*V*_p_ (cm^3^·g^−1^)	*d*_p_ (nm)
NIP	182.24 ± 3.24	0.396	7.62
MIP-CDHB	247.52 ± 2.12	0.495	10.15
MIP-ZEA	251.25 ± 1.96	0.504	11.24

**Table 10:** Imprinting effect of MIPs performed by binding experiments of ZEA.

Polymer Code	Q_MIP_	Q_NIP_	α
NIP	-	38.18	
MIP-CBHB	48.40	-	1.27
MIP-ZEA	47.85	-	1.25

## Supplementary Materials

Supplementary materials can be found at https://www.mdpi.com/1422-0067/20/7/1588/s1.
