# Supplementary material for: Towards A New Approach for the Description of Cyclo–2,4-Dihydroxybenzoate, A Substance Which Effectively Mimics Zearalenone in Imprinted Polymers Designed for Analyzing Selected Mycotoxins in Urine
Source: Int J Mol Sci. 2019 Mar 29;20(7):1588. doi: 10.3390/ijms20071588 (PMC6479585; doi:10.3390/ijms20071588)
Supplement: Supplementary file 1 [file ijms-20-01588-s001.pdf]

Table S1. A comparison of DFT geometry of conformers B3LYP/6-311++G**l with the adopted method and basis; with our X-ray data.

		EXP		DFT			
		Mol 1	Mol 2	K-1	K-2	K-3	K-4
Bond distances (Å)	C1-C6	1.397(2)	1.410(2)	1.408	1.405	1.405	1.408
	C1-C2	1.402(2)	1.406(3)	1.418	1.419	1.421	1.418
	C1-C7	1.453(2)	1.458(3)	1.466	1.478	1.470	1.466
	C2-O1	1.3528(19)	1.357(2)	1.342	1.350	1.343	1.342
	C2-C3	1.379(2)	1.379(2)	1.398	1.396	1.396	1.398
	C3-C4	1.376(2)	1.383(2)	1.389	1.388	1.387	1.389
	C4-O2	1.3625(19)	1.361(2)	1.362	1.363	1.363	1.362
	C4-C5	1.390(2)	1.386(2)	1.404	1.404	1.405	1.404
	C5-C6	1.358(2)	1.373(2)	1.379	1.381	1.382	1.379
	O21B-C26	-	1.383(6)				
	C7-O3	1.2322(19)	1.231(2)	1.231	1.210	1.230	1.231
	C7-O4	1.3310(19)	1.327(2)	1.339	1.371	1.337	1.339
	O4-C8	1.4725(19)	1.476(2)	1.467	1.470	1.474	1.467
	C8-C19	1.508(2)	1.509(3)	1.531	1.531	1.534	1.531
	C8-C9	1.514(2)	1.511(3)	1.534	1.534	1.537	1.534
Bond angles (°)	C6-C1-C2	117.51(16)	118.65(18)	118.51	118.08	118.42	118.51
	C6-C1-C7	122.54(16)	122.1(2)	119.13	116.66	122.29	122.37
	O1-C2-C3	116.76(16)	115.0(2)	122.37	116.20	117.49	117.50
	C3-C2-C1	120.63(17)	120.39(19)	119.81	120.02	120.05	119.81
	C4-C3-C2	119.89(17)	120.1(2)	120.06	120.02	119.95	120.06
	O2-C4-C3	122.67(17)	122.0(2)	122.17	117.20	117.19	122.17
	O2-C4-C5	116.74(17)	117.69(19)	116.87	122.10	121.90	116.87
	C3-C4-C5	120.60(17)	120.27(19)	120.95	120.70	120.91	120.95
	C6-C5-C4	119.09(17)	120.5(2)	118.89	118.86	119.06	118.89
	C5-C6-C1	122.26(17)	120.1(2)	121.78	122.08	121.61	121.78
	C25-C26-O21B	-	115.7(3)				
	O21B-C26-C21	-	124.2(3)				
	O3-C7-O4	122.34(17)	122.8(2)	122.59	122.12	123.63	122.59
	O3-C7-C1	123.79(16)	123.1(2)	123.29	125.05	122.90	123.29
	O4-C7-C1	113.85(16)	114.1(2)	114.12	112.83	113.47	114.12
	C7-O4-C8	118.08(14)	120.25(16)	118.45	118.33	123.40	118.45
	O4-C8-C19	105.84(13)	108.21(15)	109.51	109.55	113.59	105.69
	O4-C8-C9	108.93(15)	105.85(15)	105.69	105.95	111.34	109.51
	C19-C8-C9	115.65(16)	115.40(18)	115.41	115.66	116.22	115.41
